# Topical Application of *Escherichia coli*-Encapsulated dsRNA Induces Resistance in *Nicotiana benthamiana* to Potato Viruses and Involves RDR6 and Combined Activities of DCL2 and DCL4

**DOI:** 10.3390/plants10040644

**Published:** 2021-03-29

**Authors:** Khouloud Necira, Mongia Makki, Eugenio Sanz-García, Tomás Canto, Fattouma Djilani-Khouadja, Francisco Tenllado

**Affiliations:** 1 Laboratory of Molecular Genetics, Immunology and Biotechnology, Faculty of Sciences of Tunis, University of Tunis El Manar, Manar II, Tunis 2092, Tunisia; khouloud.necira@fst.utm.tn (K.N.); mongiamaki@gmail.com (M.M.); fattouma.djilani@yahoo.fr (F.D.-K.); 2 Departamento de Biotecnología Microbiana y de Plantas, Centro de Investigaciones Biológicas Margarita Salas, CSIC, 28040 Madrid, Spain; eugeniosanz97@gmail.com (E.S.-G.); tomas.canto@cib.csic.es (T.C.)

**Keywords:** RNAi-mediated silencing, topically applied dsRNA, encapsulated dsRNA, Dicer-like proteins, RNA-directed RNA polymerase 6

## Abstract

Exogenous application of double-stranded RNAs (dsRNAs) for inducing virus resistance in plants represents an attractive alternative to transgene-based silencing approaches. However, improvement of dsRNA stability in natural conditions is required in order to provide long-term protection against the targeted virus. Here, we tested the protective effect of topical application of *Escherichia coli*-encapsulated dsRNA compared to naked dsRNA against single and dual infection by *Potato virus X* expressing the green fluorescent protein (PVX-GFP) and Potato virus Y (PVY) in *Nicotiana benthamiana*. We found that, in our conditions, the effectiveness of *E. coli*-encapsulated dsRNA in providing RNAi-mediated protection did not differ from that of naked dsRNA. dsRNA vaccination was partly effective against a dual infection by PVX-GFP and PVY, manifested by a delay in the expression of the synergistic symptoms at early times after inoculation. Using PVX-GFP as a reporter virus together with a suite of RNAi knockdown transgenic lines, we have also shown that RNA-directed RNA polymerase 6 and the combined activities of DICER-like 2 (DCL2) and DCL4 act to promote efficient resistance to virus infection conferred by topical application of dsRNA in *N. benthamiana*. Our results provide evidence that exogenous dsRNA molecules are processed by the RNA silencing pathways commonly used by the host in response to virus infection.

## 1. Introduction

RNA interference (RNAi) is an RNA-mediated regulatory mechanism conserved in most eukaryotes, consisting of the sequence-specific degradation of targeted RNAs, guided by complementary small RNAs (sRNAs) [1]. Besides its crucial activity in regulating growth and development, RNAi plays a critical role in host defense against pathogens, including plant viruses [2,3]. RNAi against viruses is initiated by viral double-stranded (ds) RNA molecules that derive either from viral replication intermediates or from hairpin RNAs. The dsRNA molecules are recognized by specific RNase type III-like enzymes designated in plants as Dicer-like proteins (DCL), which cleave the long dsRNA fragments into sRNA duplexes of 21–25 nucleotides (nt) in size [4]. Then, the sRNAs bind to Argonaute protein family members that constitute the core of the RNA-induced silencing complexes (RISC).

In *Nicotiana benthamiana*, four DCL proteins have been described, with close homology with the ones in *Arabidopsis thaliana* [5]. DCL1 is involved in the micro-RNA biogenesis pathway and produces 21 nt sRNAs from miRNA precursors. DCL1 has also been proposed to affect DNA methylation and contribute to the biogenesis of DNA virus sRNAs by other DCLs [6]. DCL2 generates 22 nt long sRNAs and is mainly involved in antiviral defense. DCL2 participates in the production of secondary sRNAs, which trigger the phenomenon of transitivity [7]. In addition, there is mounting evidence that its role in antiviral defense is masked by DCL4 activity [8]. DCL3 is involved in the production of 24 nt sRNAs related to the RNA-directed DNA methylation pathway [9]. DCL4 generates 21 nt sRNAs and is the primary DCL component of antiviral defense against RNA viruses, as DCL4 mutants are more susceptible to different viruses [10]. In particular, it was demonstrated that DCL4 plays a critical role in the inhibition of *Potato virus X* (PVX) in inoculated leaves, while both DCL4 and DCL2 are required to prevent PVX infection in upper leaves in Arabidopsis [11]. Although the role of each DCL protein seems to be rather specific, redundancy between these pathways has been proposed [10].

RNAi-mediated viral resistance also requires host RNA-directed RNA polymerases (RDRs) to produce secondary viral sRNA molecules, thus amplifying the antiviral response [12,13]. During this amplification phase, viral RNA fragments generated by primary sRNA-mediated cleavage serve as a template for the host RDRs to produce de novo dsRNA, which is subsequently processed into 21, 22 and 24 nt secondary sRNAs by DCL4, DCL2 and DCL3, respectively [14]. Indeed, accumulation of PVX was markedly increased in the *N. benthamiana* RDR6 mutant, indicating that amplification of RNA silencing plays an important role in antiviral defense [15].

Since the discovery of the RNAi-mediated antiviral defense mechanism, a number of transgene-based silencing approaches have been developed with the aim to protect plants against viruses [16]. However, not only are these transgenic technologies time-consuming, expensive and require efficient plant transformation protocols, but they also are prone to significant regulation and social acceptance issues. A non-transgenic approach based on exogenous application of dsRNA for inducing virus resistance in plants represents an attractive and promising alternative [17]. New techniques for dsRNA large-scale production have recently emerged [18], and the topical application of these molecules for plant protection against viruses i.e., dsRNA vaccination, has been achieved in many cases with satisfactory results [19,20,21]. The analysis of sRNAs populations in dsRNA-treated plants by high-throughput sequencing (HTS) have confirmed that topically applied dsRNA is recognized and processed by the endogenous RNAi machinery. In several cases, the size distribution of sRNAs indicated that even if the amount of 21 and 22 nt sRNAs was slightly higher than other sRNAs, no well-defined peaks related to a particular size were observed [22,23,24]. Thus, a number of questions remain regarding the processing of exogenous applied dsRNA into sRNAs by DCL proteins, and the identity of the DCLs involved in antiviral protection triggered by topical application of dsRNA.

Soon it became apparent that a clear drawback to harnessing the potential of topical application of RNAi for virus resistance was the instability of the applied dsRNA to withstand environmental conditions. Exogenous application of naked dsRNA [25,26,27] or spraying of *Escherichia coli* expressed dsRNA [28,29] was demonstrated to protect plants only up to five days post-treatment, whereas loading dsRNA onto clay nanosheets, which facilitates sustained release of dsRNA, increased dsRNA stability and efficacy up to 20 days post-spray [22]. Other authors have developed methods for stabilizing the dsRNA within *Coryne bacterium glutamicum* cells by sterilizing the dsRNA-expressing bacterial cells with alcohols [30]. It was speculated that the encapsulation of the target dsRNA inside the bacterial cell wall could protect the internal dsRNA against, for example, exogenous RNases and UV light. Thus, improvement of dsRNA stability in natural conditions is required in order to provide long-term protection against the targeted virus.

Infection of plants by multiple viruses is a common phenomenon in nature, and a number of plant diseases are attributed to a synergistic interaction between them [31]. Synergy often is manifested by a remarkable increase in both symptom expression and virus accumulation as compared with single infection. Many synergistic interactions involve a member of the potyvirus group and viruses belonging to other families. The best-studied synergistic interaction involves *Potato virus Y* (PVY) and PVX in *Nicotiana* species [32,33]. Dual infection of *N. benthamiana* plants with PVX and PVY produces a disease characterized by a severe veinal necrosis of the systemically infected leaves that lead to the death of the plant. Up to now, exogenous application of dsRNA for inducing resistance against a multiple infection in plants has not been addressed.

In this study, we tested the protective effect of topical application of *E. coli*-encapsulated dsRNA compared to naked dsRNA against single and dual infection by PVX expressing the green fluorescent protein (PVX-GFP) and PVY in *N. benthamiana*. In addition, PVX-GFP was used to evaluate the differential contribution of the four *N. benthamiana DCL* and *RDR6* genes, core components of the host RNA silencing pathways, in local RNAi mediated by topical application of dsRNA. Our results show that, while DCL1 and DCL3 do not contribute significantly to the inhibition of virus multiplication in inoculated tissues, RDR6 and the combined activity of DCL2 and DCL4 play a major role in RNAi-based silencing induced by topically applied dsRNA in *N. benthamiana*.

## 2. Results

### 2.1. E. coli-Encapsulated dsRNA Provides Protection against PVY

**Figure 1:**
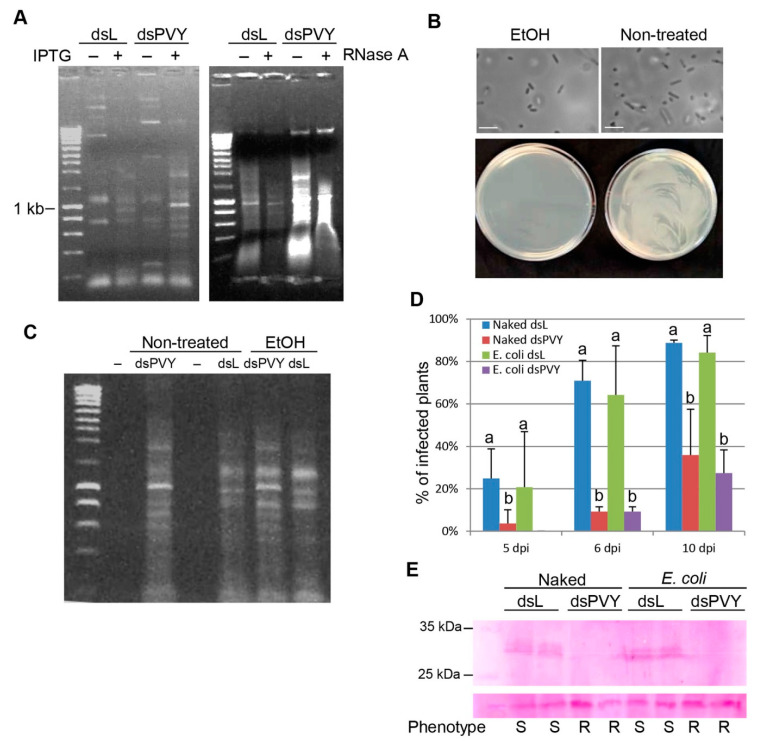
Bacterially produced dsRNA interferes with *Potato virus Y* (PVY) infection. (**A**) HT115(DE3) cultures transformed with either L4440 expressing a dsRNA consisting of 902 bp of the PVY CP gene (dsPVY) or with the empty plasmid (dsL) were induced with IPTG and processed for total nucleic acid. Samples were resolved by electrophoresis on 1% agarose gel before (left panel) or after treatment with RNase A (right panel). NZYDNA Ladder III was used as DNA markers. (**B**) Bacterial cells were non-treated or treated with ethanol 70% and imaged using a charge coupled device (CCD) camera (160×, upper panel). Scale bar denotes 2 µm. Suspension cells were plated on Luria–Bertani (LB) agar for testing survival (bottom panel). (**C**) Nucleic acids were extracted from treated and non-treated cells expressing dsPVY and dsL and electrophoresed on 1% agarose gel. (**D**) *Nicotiana benthamiana* plants were inoculated with mixtures of PVY combined with nucleic acid extracts prepared from *E. coli* accumulating the dsPVY (naked dsPVY) or *E. coli*-encapsulated dsPVY (*E. coli* dsPVY). Total nucleic acid extracted from *E. coli* expressing the dsL or *E. coli*-encapsulated dsL were used as controls. The mean percentage ± standard deviations of inoculated plants showing systemic symptoms in three independent experiments was monitored at different days post-inoculation (dpi). Statistical comparisons between means were made by employing Scheffé’s multiple range test (*p* < 0.05). Different letters indicate significant differences at *p* values of 0.05. (**E**) Western blot analysis of protein extracts derived from upper leaves of plants susceptible (S) or resistant (R) at 10 dpi, using antibodies against PVY CP. The lower panel shows the Ponceau S-stained membrane after blotting, as the control of loading.

*Escherichia coli* strain HT115(DE3) was chosen to take advantage of an isopropyl-β-D-thiogalactopyranoside (IPTG)-inducible T7 RNA polymerase gene contained within a stable insertion of a modified lambda prophage λDE3 [34]. This strain is deficient for RNase III, an enzyme that normally degrades a majority of dsRNAs in the bacterial cell, thereby improving the accumulation of extended dsRNA duplexes. We cloned a cDNA fragment containing the entire coat protein (CP) gene plus flanking sequences (902 bp) of PVY into L4440, a plasmid vector which has two convergent T7 promoters flanking the multiple cloning site [29,34]. As a negative control, HT115(DE3) harboring the L4440 empty vector was used. Upon IPTG induction (10 µM), a prominent band corresponding in size to the PVY CP dsRNA (dsPVY) was detected in bacterial nucleic acid extracts by agarose gel analysis and confirmed by resistance to RNase A, an enzyme that specifically degrades single-stranded RNA at high salt conditions (Figure 1A). A dsRNA species of ca 850 bp derived from the L4440 plasmid backbone sequences was induced in the HT115 control (dsL). It has been reported that RNA products synthesized by T7 polymerase re-bind to the polymerase and self-prime generation of hairpin duplexes [35].

As a method of providing encapsulated dsRNA for practical use, we attempted to treat dsRNA-producing cells with ethanol. Generally, an ethanol concentration of around 70% was effective for the sterilization of microorganisms [30]. Thus, we investigated whether such conditions could also be applied to the sterilization of *E. coli* HT115(DE3) accumulating the dsPVY. Not one microbial colony was detected on agar medium plates, indicating that the survival rate of the cells was negligible (Figure 1B). Charge-coupled device (CCD) imaging indicated that cell wall integrity was preserved in ethanol-treated HT115 cells. Next, the stability of the dsRNA within the sterilized cells was examined (Figure 1C). Total RNA from both dsPVY- and dsL HT115 cells was extracted from ethanol-treated and non-treated samples and subjected to agarose gel analysis to examine the integrity of the target dsRNA. No significant degradation of dsRNA occurred in ethanol-treated samples from dsPVY- and dsL-producing HT115 cells.

The effectiveness of *E. coli*-encapsulated dsRNA, compared to that of naked dsRNA, in providing RNAi-mediated protection against PVY was tested by systemic infection assays. *N. benthamiana* plants were inoculated with mixtures of PVY and either nucleic acid extracts prepared from HT115 accumulating the dsPVY, or sterilized cells containing the dsPVY. Total nucleic acids extracted from HT115 expressing the dsL or sterilized HT115 cells containing dsL were used as controls. In three independent experiments performed within a minimum of three weeks from each other, a total of 44 naked dsPVY and 44 encapsulated dsPVY-treated plants (20, 15 and 9 individuals, respectively) together with 27 plants (10, 9 and 8 individuals, respectively) from each control treatment were tested. The mean percentage of plants showing symptoms of stunting and curling was 36, 27, 89 and 84% for naked dsPVY, encapsulated dsPVY, naked dsL and encapsulated dsL treatments respectively, at 10 days post-inoculation (dpi) (Figure 1D) and remained unchanged until the end of the plant life cycle. The number of plants showing PVY symptoms in treatments with encapsulated and naked dsPVY differed significantly from treatments with encapsulated and naked dsL, respectively, whereas a slight but not significant difference in protection was observed between plants treated with encapsulated and naked dsPVY. A subset of protected *N. benthamiana* plants from each treatment was analyzed at 10 dpi by Western blot using antibodies against PVY CP, to confirm visual symptom evaluation (Figure 1E). Taken together, these results show that topical application of *E. coli*-encapsulated dsRNAs homologous to the CP gene can suppress or constrain viral multiplication and protects *N. benthamiana* plants from PVY infection. The effectiveness of *E. coli*-encapsulated dsRNA to provide RNAi-mediated protection by inhibiting or delaying symptom development did not differ greatly from that of naked dsRNA.

### 2.2. dsRNA Provides Protection against PVX Infection Foci

**Figure 2:**
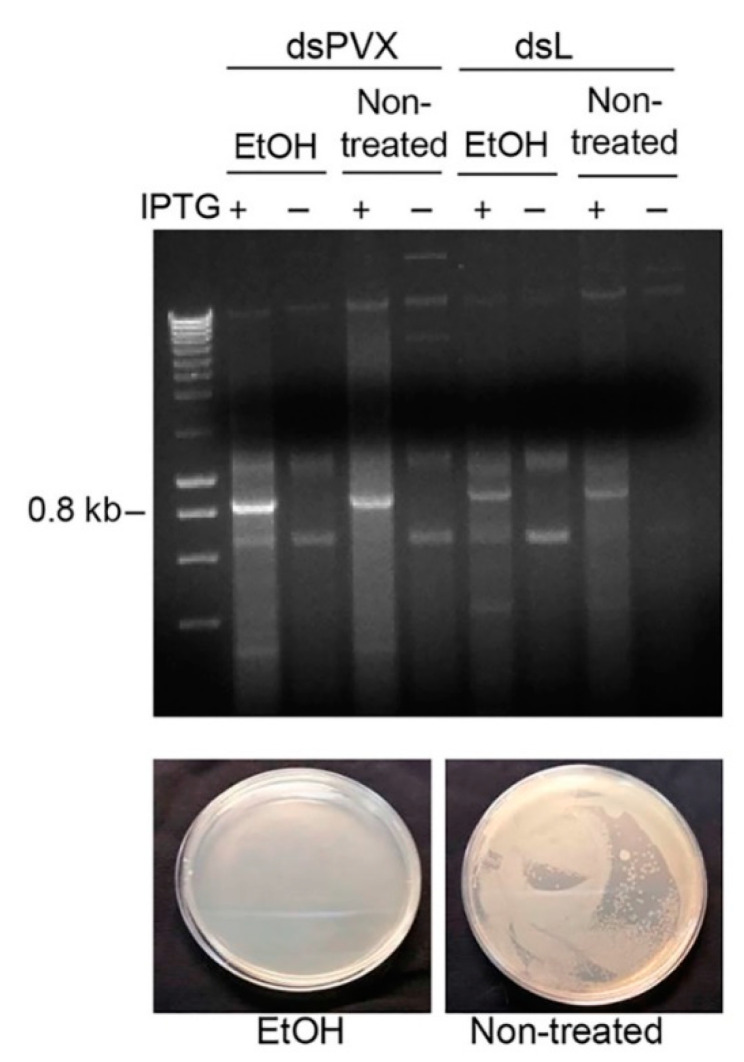
Production of *Potato virus X* (PVX) dsRNA in an *E. coli* strain deficient for RNase III. HT115(DE3) was transformed with either L4440 expressing a dsRNA consisting of 819 bp of the PVX CP gene (dsPVX) or with the empty plasmid (dsL). Bacterial cultures induced with IPTG were non-treated or treated with ethanol 70%, and processed for total nucleic acid. Samples were resolved by electrophoresis on 1% agarose gel. NZYDNA Ladder III was used as DNA markers. Bacterial cells were plated on LB agar for testing survival (bottom panels).

To test the effectiveness of *E. coli*-encapsulated dsRNA to provide RNAi-mediated protection against PVX, a cDNA fragment containing the entire CP gene plus flanking sequences (819 bp) of PVX was cloned into L4440. Upon IPTG induction, a prominent band corresponding in size to the PVX CP dsRNA (dsPVX) was detected in nucleic acid extracts derived from non-treated and ethanol-treated HT115 cells expressing dsPVX by agarose gel analysis (Figure 2).

**Figure 3:**
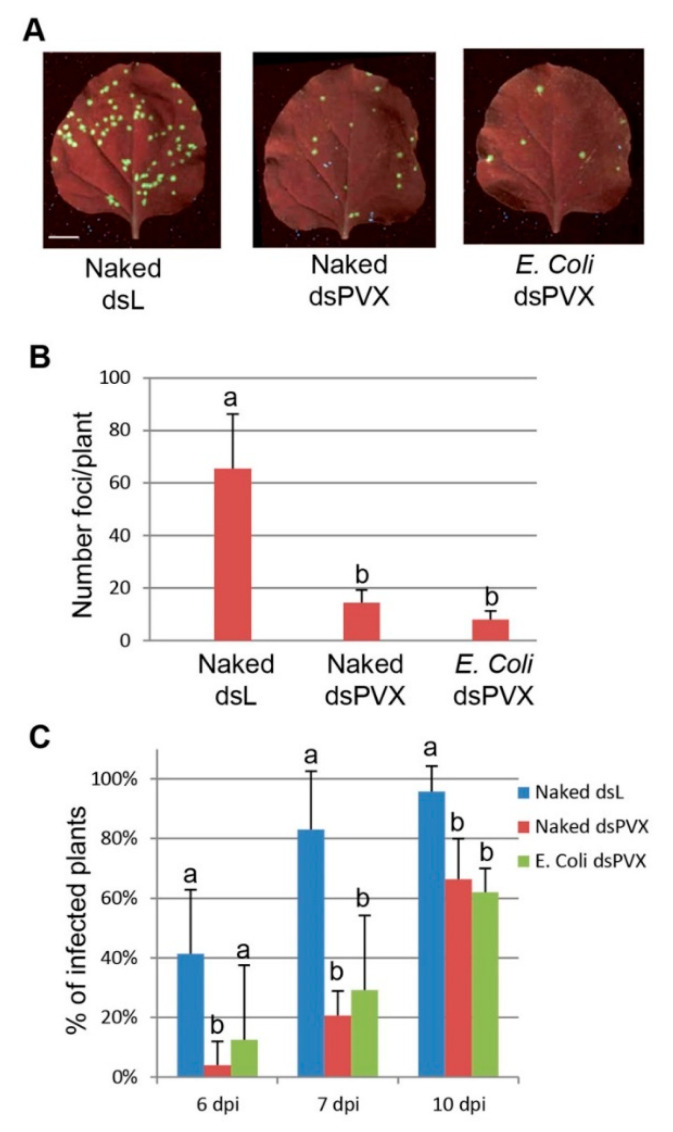
Bacterially produced dsRNA interferes with PVX-GFP infection. *Nicotiana benthamiana* plants were inoculated with mixtures of PVX-GFP combined with nucleic acid extracts prepared from *E. coli* accumulating the dsPVX (naked dsPVX) or *E. coli*-encapsulated dsPVX (*E. coli* dsPVX). The total nucleic acid extracted from *E. coli* expressing the dsL was used as the control. (**A**) Representative inoculated leaves were examined under UV light at 4 days post-inoculation (dpi). Scale bar denotes 1 cm. (**B**) Mena numbers ± standard deviations of infection foci on inoculated leaves of plants with the different treatments at 4 dpi. Different letters indicate significant differences determined by employing Scheffé’s multiple range test (*p* < 0.05). (**C**) Themean percentage ± standard deviations of inoculated plants showing systemic symptoms were monitored at different dpi. Different letters indicate significant differences determined by employing Mann–Whitney U test with a Bonferroni correction for multiple comparisons of alfa = 0.016 (*p* < 0.016).

*N. benthamiana* plants were inoculated with mixtures of PVX expressing the green fluorescent protein (PVX-GFP) and either nucleic acid extracts prepared from HT115 accumulating the dsPVX, or sterilized HT115 cells containing the dsPVX. The total nucleic acid extracted from HT115 expressing the dsL was used as the control. A total of 24 plants from each treatment were tested in four independent experiments performed within a minimum of two weeks from each other (six individuals per experiment), and the subsequent viral infection was monitored by detecting GFP foci in the inoculated leaves. At 4 dpi, there were fewer fluorescent spots or infection foci on the inoculated leaves of treatments with encapsulated and naked dsPVX than with naked dsL (8- and 4.5-fold, respectively), implying that the presence of dsPVX at least partially blocked PVX infection (Figure 3A,B).

Most of the *N. benthamiana* plants (83%) inoculated with PVX-GFP plus dsL showed typical symptoms of chlorosis and GFP fluorescence in systemic leaves at 7 dpi. At the same time point, only 29% and 20% of plants treated with PVX-GFP plus either encapsulated or naked dsPVX appeared symptomatic, respectively. At later observation dates (10 dpi), the rate of symptomatic plants in treatments with encapsulated and naked dsPVX reached 62% and 66% respectively and remained unchanged until the end of the plant life cycle (Figure 3C).

**Figure 4:**
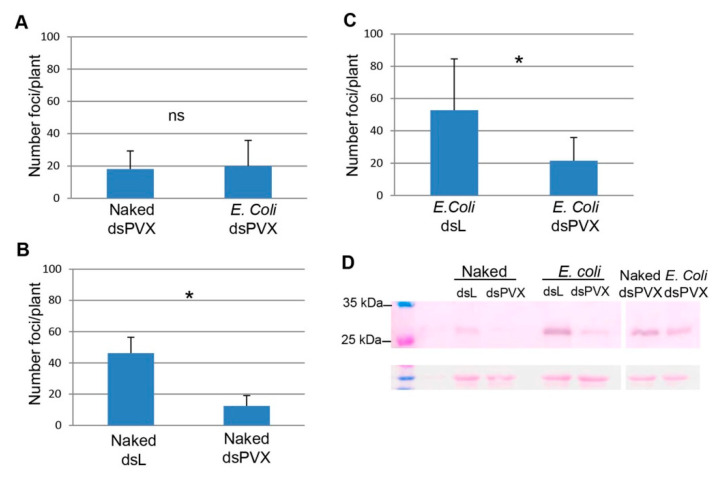
Effectiveness of encapsulated and naked dsPVX to induce local protection against PVX-GFP. The mean numbers ± standard deviations of infection foci on the inoculated leaves of plants that received different treatments are shown. (**A**) One half of the leaf was inoculated with PVX-GFP plus E. coli-encapsulated dsPVX (E. coli dsPVX) while the opposite half was inoculated with the virus and naked dsPVX. (**B**) One half of the leaf was inoculated with PVX-GFP plus naked dsPVX while the opposite half was inoculated with the virus and naked dsL. (**C**) One half of the leaf was inoculated with PVX-GFP plus E. coli-encapsulated dsPVX while the opposite half was inoculated with the virus and E. coli-encapsulated dsL (E. coli dsL). Asterisks indicate significant differences between treatments (Student’s t-test, *p* < 0.05). Ns: not significant. (**D**) Western blot analysis of plant extracts derived from inoculated leaves at 4 days post-inoculation, using antibodies against PVX CP. The upper right panel represents a longer time of exposure. The lower panel shows the Ponceau S-stained membrane after blotting, as a control of loading. * significant.

In a different experiment using 14 plants per treatment, the effectiveness of encapsulated vs. naked dsPVX to induce local protection against PVX-GFP was compared directly in the same leaves. One half of the leaf was inoculated with PVX-GFP plus encapsulated dsPVX while the opposite half was inoculated with the virus and naked dsPVX. For controls, one half of the leaf was inoculated with PVX-GFP plus either encapsulated or naked dsPVX while the opposite half was inoculated with the virus and encapsulated or naked dsL, respectively. Again, the number of GFP foci was clearly reduced in plants treated with encapsulated and naked dsPVX compared to controls at 4 dpi (Figure 4B,C). However, there was no difference in the number of GFP foci between encapsulated and naked dsPVX-treated leaves, implying a similar interference effect against PVX-GFP (Figure 4A). Western blot analysis showed that PVX CP accumulated less in treatments with encapsulated and naked dsPVX compared to encapsulated and naked dsL, respectively, while similar levels of CP were detected in treatments with encapsulated and naked dsPVX (Figure 4D).

### 2.3. A Combination of dsRNAs Partially Constraints Dual Infection by PVX and PVY

**Figure 5:**
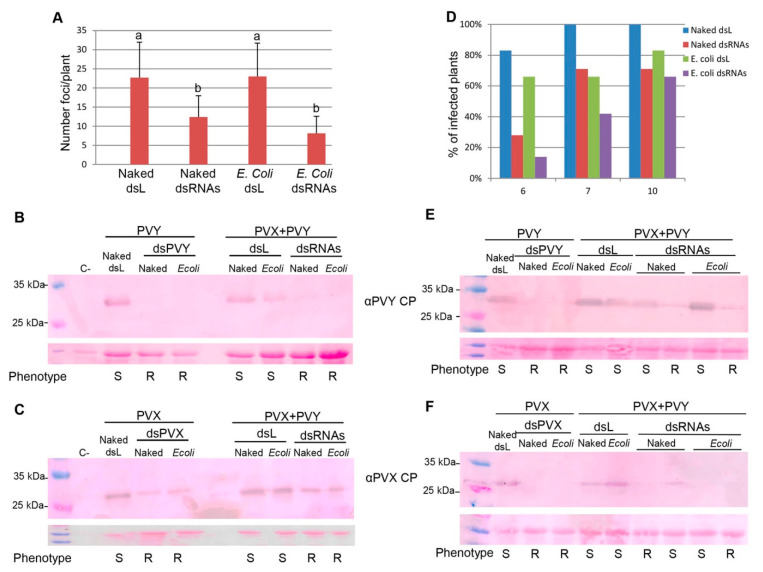
Bacterially produced dsRNA delays dual infection by PVY and PVX-GFP. *N. benthamiana* plants were inoculated with PVX-GFP plus PVY combined with *E. coli*-encapsulated dsPVX and dsPVY (*E. coli* dsRNAs), PVX-GFP plus PVY combined with naked dsPVX and dsPVY (naked dsRNAs), PVX-GFP plus PVY combined with *E. coli*-encapsulated dsL, and PVX-GFP plus PVY combined with naked dsL. For controls, plants were singly infected with PVX-GFP or PVY together with the corresponding dsRNA targeting the homologous virus. (**A**) Mean numbers ± standard deviations of PVX-GFP foci on doubly inoculated leaves of plants with the different treatments at 4 days post-inoculation (dpi). Different letters indicate significant differences determined by employing Scheffé’s multiple range test (*p* < 0.05). Western blot analysis of plant extracts derived from inoculated leaves at 4 dpi, using antibodies against PVY CP (**B**) and PVX CP (**C**). Plants susceptible (S) or resistant (R) to virus infection at 6 dpi are indicated. Lane C corresponds to the extract from a non-infected plant. (**D**) The percentage of inoculated plants showing systemic symptoms was monitored at different dpi. Western blot analysis of plant extracts derived from upper leaves of plants susceptible (S) or resistant (R) at 7 dpi, using antibodies against PVY CP (**E**) and PVX CP (**F**). The lower panel shows the Ponceau S-stained membrane after blotting, as a control of loading.

To test the effectiveness of *E. coli*-encapsulated dsRNA in providing RNAi-mediated protection against double infection by PVX-GFP and PVY, *N. benthamiana* plants were inoculated with four different treatments, i.e., PVX-GFP plus PVY combined with encapsulated dsPVX and dsPVY, PVX-GFP plus PVY combined with naked dsPVX and dsPVY, PVX-GFP plus PVY combined with encapsulated dsL, and PVX-GFP plus PVY combined with naked dsL. For controls, plants were singly infected with either PVX-GFP or PVY, together with the corresponding dsRNA targeting the homologous virus. A total of 30 plants per treatment were doubly inoculated in three independent experiments, and subsequent PVX-GFP infection was monitored by detecting GFP foci in the inoculated leaves. At 4 dpi, the number of infection foci on leaves treated with encapsulated and naked viral dsRNAs was significantly lower than those on plants treated with encapsulated and naked dsL, respectively (Figure 5A). Samples were randomly taken from four inoculated leaves derived from different plants and assayed by Western blot analysis at 4 dpi (Figure 5B,C). The PVX CP accumulated less in treatments with encapsulated and naked viral dsRNA compared to treatments with either encapsulated or naked dsL, respectively, whereas the PVY CP was absent in samples derived from encapsulated and naked dsRNA treatments.

In a separated experiment, systemic symptoms of chlorosis and stunting were observed in about 80% and 65% of PVX-GFP/PVY-inoculated plants treated with naked and encapsulated dsL respectively, at 6 dpi (Figure 5D); conversely, only 28% of plants treated with naked viral dsRNAs and 14% of plants treated with encapsulated viral dsRNAs appeared symptomatic. At later observation dates (10 dpi), about 70% plants treated with encapsulated and naked viral dsRNAs showed systemic symptoms of viral infection, mostly necrotic symptoms characteristics of the double infection by PVX-GFP/PVY. Samples were taken from systemic leaves derived from symptomatic and asymptomatic plants and assayed by Western blot analysis at 7 dpi (Figure 5E,F). The PVX-GFP and PVY CPs accumulated less in asymptomatic plants of treatments with encapsulated and naked viral dsRNAs compared to encapsulated and naked dsL, respectively. Taken together, these results show that the combined topical application of dsRNAs homologous to the CP genes of PVX-GFP and PVY partially constrains viral multiplication in doubly inoculated plants, which is manifested by a delay in the expression of the synergistic symptoms in N. benthamiana. Again, the effectiveness of E. coli-encapsulated dsRNAs in providing RNAi-mediated protection against dual infection by PVX-GFP-PVY did not differ from that of naked dsRNAs.

### 2.4. Involvement of DCL2, DCL4 and RDR6 in RNAi Induced by Topically Applied dsRNA

**Figure 6:**
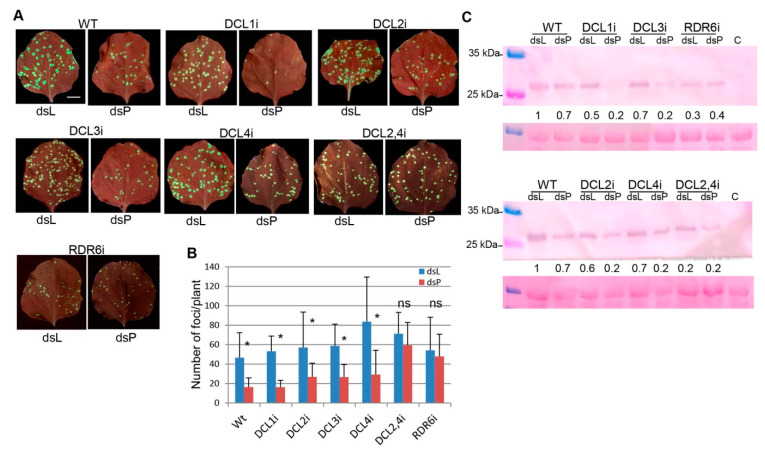
Role of DCL and RDR6 proteins in RNAi induced by topically applied dsRNA. Wt and DCL1i, DCL2i, DCL3i, DCL4i, DCL2/4i and RDR6i mutant lines were inoculated with mixtures of PVX-GFP and either naked dsPVX (dsP) or dsL. (**A**) Representative inoculated leaves were examined under UV light at 4 days post-inoculation (dpi). Scale bar denotes 1 cm. (**B**) Mean values ± standard deviations of infection foci per plant counted at 4 dpi on 2 inoculated leaves for a total of 10 plants per treatment. Asterisks indicate significant differences between treatments (Student’s t-test, *p* < 0.05). Ns: not significant. Data presented were collected from two independent experiments. (**C**) Western blot analysis of plant extracts derived from inoculated leaves at 4 dpi, using antibodies against PVX CP. Lane C corresponds to the extract from a non-infected plant. The lower panel shows the Ponceau S-stained membrane after blotting, as a control of loading. The intensity of each PVX CP band was quantified by densitometry analyses, and normalized to the Ponceau S-stained band. Protein levels in Wt leaves treated with dsL are given the value of 1 and other data were calculated relative to this value. * significant.

In order to evaluate the role of DCL proteins in RNAi induced by topical application of dsRNA, we used *N. benthamiana* transgenic lines suppressed for each one of the four known Dicer-like (DCLi) genes and for a combination of DCL2 and DCL4 (DCL2/4i). DCL genes are core components of host RNA-mediated silencing pathways. Quantitative RT-PCR analyses of transgenic lines DCL1i, DCL2i, DCL3i, DCL4i and DCL2/4i have previously shown a specific reduction in the DCL1, DCL2, DCL3, and DCL4 mRNAs levels, respectively [36]. In addition, transgenic *N. benthamiana* (line RDR6i) expressing an inverted repeat fragment of its RDR6 gene that reduced the level of the endogenous transcript mRNA was also used [15]. Wt and DCL1i, DCL2i, DCL3i, DCL4i, DCL2/4i and RDR6i lines were inoculated with mixtures of PVX-GFP and either naked dsPVX or dsL. A total of 12 plants from each treatment were tested in two independent experiments, and the subsequent viral infection was monitored by detecting GFP foci in the inoculated leaves (Figure 6A). At 4 dpi, there were fewer infection foci on the inoculated leaves of Wt and DCL1i, DCL2i, DCL3i, and DCL4i lines treated with dsPVX than on the corresponding leaves treated with dsL (2.1- to 3.4-fold) (Figure 6B). Interestingly, there were no statistical differences in the number of infection foci in DCL2/4i and RDR6i lines treated with dsPVX compared to dsL. A western blot analysis showed that PVX CP tends to accumulate less in treatments with dsPVX compared to dsL in Wt and DCL1i, DCL2i, DCL3i, and DCL4i lines, while similar levels of CP were detected in treatments with dsPVX and dsL in DCL2/4i and RDR6i lines (Figure 6C).

**Figure 7:**
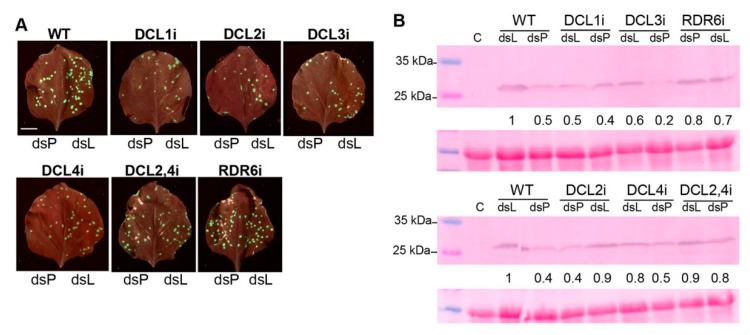
Side by side comparisons of RNAi mutants. (**A**) One half of the leaf from Wt and DCL1i, DCL2i, DCL3i, DCL4i, DCL2/4i and RDR6i mutant lines was inoculated with PVX-GFP plus naked dsPVX (dsP) while the opposite half was inoculated with the virus and naked dsL. Representative inoculated leaves were examined under UV light at 4 days post-inoculation. Scale bar denotes 1 cm. (**B**) Western blot analysis of plant extracts derived from inoculated leaves at 4 dpi, using antibodies against PVX CP. Lane C corresponds to extract from non-infected plant. The lower panel shows the Ponceau S-stained membrane after blotting, as a control of loading. The intensity of each PVX CP band was quantified by densitometry analyses and normalized to the Ponceau S-stained band. Protein levels in Wt leaves treated with dsL are given the value of 1 and other data were calculated relative to this value. Note that dsL and dsP treatments were inadvertently loaded in the reverse orientation in DCL2 and RDR6 mutants.

To confirm the above results, the effectiveness of the different DCLi and RDR6 mutants to induce local RNAi against PVX-GFP was compared directly in the same leaves. One half of the leaf was inoculated with PVX-GFP plus naked dsPVX while the opposite half was inoculated with the virus and naked dsL. Again, the number of GFP foci was clearly reduced in Wt and DCL1i, DCL2i, DCL3i, and DCL4i lines treated with naked dsPVX compared to controls at 4 dpi (Figure 7). Interestingly, the number of GFP foci did not vary greatly between dsPVX and dsL-treated halves in DCL2/4i and RDR6i lines. A western blot analysis performed on Wt and the different mutant lines confirmed similar levels of PVX CP in treatments with dsPVX and dsL in DCL2/4i and RDR6i lines. Taken together, these results show that both the RDR6 and the combined activities of DCL2 and DCL4 are major players in RNAi-mediated viral resistance induced by topical application of dsRNA.

## 3. Discussion

A major bottleneck for RNAi-based protection in crops is that naked dsRNA, when delivered on plants several days before virus inoculation, has been shown to protect plants for a limited time-window of only four to seven days [23,28,29]. It has been proposed that delivery of dsRNA using polymer nanoparticles, liposomes or bacteria, could increase efficacy in attaining a potent and long-lasting RNAi response [37]. Indeed, incorporation of dsRNA into hydroxide nanolayers, called BioClay, increased the duration of the protection against virus infection by at least 20 days [22]. Bacteria-encapsulated dsRNA has been successfully used to reduce the damage caused by the ladybird beetle and Colorado potato beetle in potatoes [30,38]. In several cases, encapsulated dsRNA was shown to confer more efficient protection against pests compared to similar treatments with naked dsRNA [38,39]. It was suggested that packaging dsRNA in a protective bacterial cell wall may have a stabilizing effect on the presence of dsRNA in the lumen of the digestive system of the insect. Here, we established that the topical application of *E. coli*-encapsulated dsRNA is also effective against infection by RNA viruses, i.e., PVX-GFP and PVY, in the model plant *N. benthamiana*. However, we found that, in our conditions, the effectiveness of *E. coli*-encapsulated dsRNA to provide RNAi-mediated protection did not differ from that of naked dsRNA. This implies that dsRNA degradation inside plant tissues is not a limiting factor affecting RNAi once access of dsRNA molecules through the plant cell wall is facilitated by the abrasive. Bacterium-mediated RNA interference, wherein live endophytic bacteria expressing dsRNA are applied to and colonize an organism to produce and facilitate the uptake of dsRNA, has been shown to be effective in insects [40]. This approach has been proposed as a potential improvement of RNAi in plants to overcome some of the limitations of more-traditional application strategies (spraying, infiltration and drenching), such as the duration and the systemic nature of the RNAi silencing effect [41].

Topical application of dsRNA against PVY provided a higher level of protection (60%) compared to that against PVX-GFP (40%). We can exclude a possible effect of the dsRNA length, since the two dsRNAs are similar in size and include the entire coding region of the CP gene. It is more likely that the reason for the lower dsRNA-mediated protection to PVX could reflect the differential accessibility of viral target RNA to degradation by RNA silencing. It has been suggested that sub-cellular compartmentalization of PVX RNA in active viral replication complexes and in specific membrane compartments [42] plays an important role in target accessibility to the machinery of RNA silencing [43]. Alternatively, we cannot exclude that the viral dsRNAs could be recognized differentially by the RNAi machinery, which would lead to different response in terms of sRNA abundance and thus, virus resistance.

Until now, RNAi-based vaccination by topical application of dsRNA has been limited to the targeting of single viruses [19,20]. Here, we established that the topically applied dsRNA was partly effective also against a dual infection by PVX-GFP and PVY, manifested by a delay in the expression of the synergistic symptoms at early times after inoculation. RNAi-mediated vaccination requires that most viral RNA penetrates into the plant cells in combination with appropriate quantities of the specific dsRNA [17,21]. The lower protection conferred by topical application of a mixture of dsRNAs directed against a dual infection compared to single infection could probably reflect the reduced probability that a single cell could receive the dsRNA and its cognate virus for each virus/dsRNA combination. It has been shown that efficient simultaneous targeting of four different tospoviruses can be achieved by using a single transgene based on the expression of minimal sized chimeric cassettes [44]. Thus, RNAi vaccination against multiple virus infection could be further improved in the future by combining RNA fragments from a series of target viruses in a single chimeric dsRNA.

In plants, RNA silencing against viral infection is regulated by proteins encoded by respective DCL and RDR gene families, among others [5]. The biogenesis of the sRNAs in plants after exogenous application of dsRNA has been documented in several studies [22,23,24]. However, the genetic basis and involvement of sRNAs in topically applied, dsRNA-mediated resistance remains poorly understood. HTS analyses of virus-inoculated plants treated with dsRNA indicated that two major sRNA size class were produced containing predominantly 21 and 22nt sRNAs, suggesting a role of DCL2 and DCL4 in the processing of dsRNA. However, plants treated only with dsRNA exhibited a different sRNA profile, suggesting that other DCLs could be also involved in the processing of dsRNA to sRNAs [22,23,24]. All four DCL proteins can potentially process the dsRNA, but this occurs in a hierarchical manner that presumably reflects the contrasted affinity of various DCLs for long dsRNA [45].

Using PVX-GFP as a reporter virus together with a suite of RNAi transgenic lines, here we have provided evidence that RDR6 and the combined activity of DCL2 and DCL4, likely along with DCL2- and DCL4-processed sRNAs, act to promote efficient resistance to virus infection conferred by topical application of dsRNA. It has been reported that different DCL family members contribute to antiviral activities against PVX and other viruses, suggesting partial functional redundancies among DCL proteins [10,11]. Indeed, the number of infection foci increased in DCLi and RDR6i mutant backgrounds inoculated with PVX-GFP plus non-specific dsRNA compared to Wt plants. Besides that, local protection against PVX-GFP induced by topical application of dsPVX was not significantly affected in single DCLi knockdown lines, whereas dsRNA-mediated virus resistance was abolished in DCL2/4i and RDR6i lines. This suggested that RDR6 activity and the combined action of DCL4 and DCL2 are involved in antiviral resistance mediated by topical application of dsRNA. DCL4 and DCL2 also redundantly mediate dsRNA-mediated RNAi used for transgene knockdown in plants [45].

The RNAi antiviral response is amplified by the actions of endogenous RDRs, which synthesize dsRNA using the viral RNA target as a template [14]. This dsRNA serves as a substrate for the production of secondary sRNAs. The RDR6 gene of *N. benthamiana* has been shown to be essential for intracellular silencing and for the production of secondary sRNAs during PVX infection [15]. So far, it has been assumed that the presence of sRNAs in tissues treated with exogenous dsRNA originate mostly from the dicing of input dsRNA (primary sRNAs) rather than from products of RDR6 activity (secondary sRNAs). Our results suggest that the RDR6 pathway and, thus, transitivity is an essential component of RNAi-mediated viral resistance induced by topical application of dsRNA. However, the complex nature of dsRNA-generating pathways makes it difficult to distinguish the relative importance of primary versus secondary sRNA synthesis in antiviral defense. Moreover, DCL4 and DCL2 have been shown to act redundantly upstream (primary sRNAs) and downstream (secondary sRNAs) of RDR6 action during RNA virus infections [12,46].

In conclusion, our results would indicate that RNAi-mediated resistance to viruses triggered by topical application of dsRNA was based on the combined activities of DCL2, DCL4 and of RDR6, thus providing evidence that exogenous dsRNA molecules are processed by the RNA silencing pathways commonly used by the host in response to virus infection. Moreover, we demonstrated that *E. coli*-encapsulated dsRNA was effective providing RNAi-mediated resistance against PVX and PVY infection. Further research is needed in order to elucidate whether delivery methods that use live dsRNA-expressing bacteria could overcome some of the current limitations of dsRNA-based technology.

## 4. Materials and Methods

### 4.1. Plasmid Construct

The complete CP gene, flanking regions of the 3′ NIb, and the beginning of the 3′ UTR of PVY (902 bp) were extracted from pBSK-CPPVY plasmid [47] using *Hind*III and *BamH*I restriction enzymes and introduced into L4440 plasmid. L4440 is a plasmid vector which has two convergent T7 promoters flanking the multiple cloning sites [34]. The complete CP coding sequence and flanking regions of PVX (819 bp) was amplified by PCR using pGR107 as template and cloned into *Xba*I and *Hind*III of L4440. The upstream primer was 5′AGCTCTAGAGATAGGGCCATTGCCGATCT3’ (nucleotides 5583 to 5604, italicized sequence corresponds to the viral sequence and the non-italicized sequence contains the *Xba*I restriction site). The downstream primer was 5′AGCAAGCTT*ACTATGAAACTGGGGTAGGCG*3´ (nucleotides complementary to 6381 to 6402; italicized sequence corresponds to the viral sequence and the non-italicized sequence contains the *Hind*III restriction site). The binary vector pGR107, which contains the infectious cDNA of PVX, was provided by D. C. Baulcombe (University of Cambridge). All the plasmids including the L4440 empty vector were sequenced to confirm the identity of the sequences. Plasmids were transformed into *E. coli* HT115(DE3) using standard CaCl_2_ transformation protocols. HT115(DE3) is an RNAase III-deficient *E. coli* strain, which was modified to express T7 RNA polymerase from an IPTG-inducible promoter [34]. The RNase III gene is disrupted by a Tn10 transposon carrying a tetracycline-resistance marker.

### 4.2. dsRNA Production

Single colonies of HT115(DE3) containing the empty L4440 or L4440 plasmid derivatives were grown at 37 °C for 16 h in a Luria–Bertani (LB) broth with ampicillin and tetracycline at a final concentration of 50 and 12.5 µg/mL, respectively. The culture was diluted 75-fold in the same medium and allowed to grow to OD_595_ = 0.5. T7 polymerase was induced by the addition of 10 µM IPTG, and the culture was incubated further with shaking for 2 h at 37 °C. After that, the culture broths were centrifuged (4000 rpm, 15 min) to collect the cells, and 1/10 volume of the buffer solution (5 mM Tris: 1 mM EDTA, pH 7.5) or buffer solution containing ethanol at 70% [*v*/*v*] was added to the cell pellet, and then cells were thoroughly re-suspended. The cell concentration was about 1.5 × 10^9^ cells/mL in the reaction tube, and the suspension was allowed to stand at RT for 20 min. After this treatment, the cell suspension was centrifuged to remove the supernatant and re-suspended in buffer solution to yield the *E. coli*-encapsulated dsRNA. *E. coli* cells were imaged using a Zeiss Axioplan Universal microscope outfitted with a DFC 350 FX CCD camera from Leica (Cambridge, UK). Alternatively, bacterial pellets were re-suspended in 1 M ammonium acetate and the total nucleic acid was extracted after a phenol-chloroform step prior to ethanol precipitation (naked dsRNA).

To examine the survival rate of ethanol-treated cells, 0.1 mL of the suspensions containing the non-treated and the ethanol-treated *E. coli* cells were spread and cultured on an LB agar medium supplemented with ampicillin and tetracycline at 37 °C for 16 h. Evaluation of dsRNA stability in the ethanol-treated cells was performed by extracting total nucleic acids, as described above, and analyzed by agarose gel (1%) electrophoresis. The concentration of total nucleic acid in different preparations was estimated to be approximately 1 µg/µL by spectrophotometry using E_260_ = 40. Accumulation of dsRNA in bacterial extracts was confirmed by resistance to RNase A under high salt conditions (0.3 M NaCl, 0.03 M sodium citrate).

### 4.3. Plant Material and Virus Inoculation

*N. benthamiana* plants were maintained in controlled growth chambers with a photoperiod of 16/8 h (day/night) and a daylight intensity of ~2500 luxes. *N*. *benthamiana* plants which contain hairpins to decrease endogenous DCL1, DCL2, DCL3, DCL4, DCL2,4 and RDR6 transcripts were described by Dadami et al. [36] and Schwach et al. [15], respectively.

A Scottish ordinary variety (O) isolate from PVY was used [48] (genbank accession number AJ585196). The PVX-GFP binary vector was previously described [49]. In order to ensure the uniformity of the viral inocula in all the experiments, inoculum stocks were prepared from systemically PVY-infected *N. benthamiana* leaves and from local leaves agro-infiltrated with PVX-GFP. For this, symptomatic leaf tissue was cut in small slices (1 cm^2^), split into 200 mg aliquots and stored at −80 °C until use. Inoculations with PVY and PVX-GFP on 3- to 4-week old plantlets were performed by grinding each aliquot in sodium phosphate buffer (0.02 M, pH 7) at 10% and 5% (*w*/*v*), respectively. A 30 µL-dose of infected sap combined with naked or encapsulated dsRNA (1:2 sap:dsRNA ratio; 20 µg dsRNA) was applied to two leaves of each plant previously dusted with carborundum as an abrasive (Carlo Erba, Barcelona, Spain). In mixed infections, equal volumes of PVX plus PVY inocula were inoculated in combination with either naked dsRNA or encapsulated dsRNA.

### 4.4. Protein Gel Blot Analysis

Total proteins were extracted by grinding leaf disks of 25 mm in diameter with a pestle, and mixed with five volumes of extraction buffer (0.1 M Tris-HCl PH 8, 10 mM EDTA, 0.1 M LiCl, 1% β-mercaptoethanol and 1% SDS). Samples were boiled and fractionated in 15% SDS-PAGE gels. For the detection of PVX, a commercial rabbit antibody was used (No. 070375/500; Loewe Biochemica GmbH, Sauerlach, Germany). The detection of PVY was performed using a rabbit polyclonal antiserum (1:1000 dilution) [47]. Blotted proteins were detected using commercial secondary antibodies and Sigma Fast^TM^BICP/NBT substrate tablets (SIGMA Aldrich, Saint Louis, MO, USA). The number of PVX-GFP-derived fluorescence was assessed with a Black Ray^®^ long wave UV lamp (UVP, Upland, CA, USA). Densitometric analyses of blotted protein bands were performed using the public domain software ImageJ (National Institutes of Health website, image processing and analysis in java).

### 4.5. Statistical Analysis

All statistical analyses were performed using the statistical software IBM SPSS Statistics v.25 (IBM Corp). For each experiment, samples were assessed for normality via the Shapiro–Wilk test, and for equality of variances using Levene’s test. For experiments with approximately normally distributed samples of equal variance, one-way ANOVA followed by Scheffé’s post hoc test was performed. Otherwise, a nonparametric Mann–Whitney U test was employed, with the Bonferroni correction for multiple comparisons between samples applied. For comparisons between pairs of means, Student’s *t*-test was employed [43].

## Data Availability

The data presented in this study are available within the article.
